# Performance Research of Ultra-High Performance Concrete Incorporating Municipal Solid Waste Incineration Fly Ash

**DOI:** 10.3390/ma18194623

**Published:** 2025-10-07

**Authors:** Fengli Liu, Yize He, Junhua Liu, Feiyang Zhang, Xiaofei Hao, Chang Liu

**Affiliations:** 1 School of Civil Engineering and Architecture, Henan University, Kaifeng 475004, China; zhangfeiyang@henu.edu.cn; 2 School of Civil Engineering and Architecture, Kaifeng University, Kaifeng 475004, China; 15037817250@163.com; 3 Zhengzhou Institute of Multipurpose Utilization of Mineral Resources, Chinese Academy of Geological Sciences, Zhengzhou 450006, China; goodpaper@cug.edu.cn; 4 Liaoning Watson Geotechnical High-tech Co., Ltd., Shenyang 110167, China; 16613786700@163.com

**Keywords:** municipal solid waste incineration fly ash (MSWIFA), ultra-high performance concrete (UHPC), microscopic characterization, durability, heavy metal solidification, environmental assessment, economic assessment

## Abstract

Waste management poses escalating threats to environmental sustainability, particularly with municipal solid waste (MSW) growth. Incineration, a widely adopted method for reducing waste volume, produces millions of tons of municipal solid waste incineration fly ash (MSWIFA) each year. Despite its high toxicity and classification as a hazardous solid waste, its ultrafine particle size and pozzolanic activity offer potential for its use in construction materials. In this study, MSWIFA was used to replace 6%, 12%, 18% and 24% of cementitious materials, and the effect of MSWIFA substitution rate on the workability, mechanical properties, microstructure, and durability of UHPC was studied. Furthermore, the study assessed the solidification capacity of the UHPC for heavy metal ions and quantitatively analyzed its eco-economic benefits. The results show that, under standard curing conditions, substituting 12% of cementitious materials with MSWIFA significantly modified UHPC hydration, shortened setting time, reduced fluidity, and increased wet packing density. The 28-day compressive strength reached 134.63 MPa, comparable to the control group. Concurrently, fluidity, durability, and heavy metal leaching all met the required standards, with energy consumption reduced by 14.86%, carbon emissions lowered by 12.76%, and economic costs decreased by 6.41%. This study provides a feasible solution for recycling MSWIFA into non-hazardous concrete, facilitating sustainable hazardous waste management and mitigating heavy metal-related environmental pollution.

## 1. Introduction

With accelerated urbanization and population growth, global municipal solid waste (MSW) production has surged to over 2 billion tons annually [1]. It is projected to increase by 70%, reaching 3.4 billion tons by 2050 [2], imposing severe environmental burdens. Incineration reduces MSW volume by 85–90% and mass by 70–90%, making it a primary MSW treatment method worldwide [3,4]. However, municipal solid waste incineration fly ash (MSWIFA) generated during this process contains toxic substances such as dioxins, heavy metals (Pb, Cu, Zn Cr and Cd), and chlorides. Consequently, MSWIFA is classified as hazardous solid waste in many countries [5]. Conventional disposal methods for MSWIFA include its utilization as cement production [6], concrete manufacturing [7], alkali-activated binders [8,9,10,11] and subgrade materials [12]. Non-resource treatments involve cement solidification or post-stabilization landfill disposal [2,13,14]. These approaches not only occupy substantial land resources, but carry long-term risks of pollutant leakage. Therefore, rational disposal and resource recycling of MSWIFA are crucial for advancing sustainable waste management.

Cement-based materials are widely used in modern construction, with an annual global consumption exceeding 40 billion tons [15,16,17,18]. Cement is the primary component of cement-based materials, with an annual global production exceeding 4 billion tons [19,20]. However, the cement industry accounts for 8% of global CO_2_ emissions [21], primarily originating from the decomposition of carbonates (such as CaCO_3_ and MgCO_3_), the combustion of fossil fuels and electricity consumption during the production process [21,22], causing significant negative environmental impacts. MSWIFA contains substantial amounts of CaO (25–40%), SiO_2_ (15–30%) and Al_2_O_3_ (8–15%) [23,24], which can chemically supplement cement clinker through hydration reactions. Furthermore, its properties are similar to supplementary cementitious materials like silica fume and slag [25,26]. However, when MSWIFA is added to ordinary concrete, the leaching concentrations of heavy metals often exceed safety limits due to the high porosity of concrete [27]. Safe utilization pathways for MSWIFA in cement-based materials require further in-depth research.

Ultra-high performance concrete (UHPC) faces limitations in engineering applications due to its high cement content, high costs, and significant carbon emissions [28,29]. Furthermore, the inherent constraint of UHPC’s low water-to-binder ratio (0.15–0.25) results in only 30–40% cement hydration, leaving 60–70% of unreacted cement particles to function merely as inert fillers [30,31], leading to substantial resource wastage. Research on low-carbon sustainable UHPC has thus emerged as a critical focus. UHPC possesses a dense microstructure, exceptional mechanical properties, and outstanding durability [28,32], offering potential advantages for immobilizing heavy metals in MSWIFA and ensuring long-term stability. Additionally, MSWIFA has specific particle size characteristics and pozzolanic reactivity. In UHPC, it serves dual functions: as a supplementary cementitious material and as a filler. Replacing cement with MSWIFA offers significant research potential for developing low-carbon sustainable UHPC.

Utilizing MSWIFA to replace cement in UHPC production has emerged as a cutting-edge approach for solid waste resource recovery and low-carbon building materials. Lv et al. [33] employed high-temperature pretreatment of MSWIFA to prepare UHPC, finding that heavy metal leaching concentrations during the initial stage met current safe limits; however, the thermal treatment significantly increased energy consumption and economic costs. Mao et al. [34] demonstrated that substituting cement with MSWIFA could reduce UHPC production energy consumption and carbon emissions by approximately 20%, though the resulting maximum compressive strength was only 103.3 MPa, which is relatively low in terms of mechanical performance. Xing et al. [35] combined MSWIFA and UHPC through chemical stabilization and physical encapsulation, significantly enhancing its mechanical properties. However, the leaching concentrations of Pb and Cd in UHPC exceeded safe limits. Theoretically, MSWIFA’s fine particle size could enable dense packing with cementitious materials, allowing partial cement substitution in UHPC. However, research on its use as a substitute for cement in the preparation of UHPC for resource utilization and sustainable management is still limited, especially regarding whether MSWIFA causes heavy metal leaching in UHPC, which remains largely unexplored and lacks a systematic assessment framework.

In view of this, this study prepared MSWIFA-UHPC by partially replacing cement with MSWIFA, systematically investigating the effects of MSWIFA replacement ratios on workability, mechanical strength, microstructure, durability, heavy metal immobilization capacity, and integrated eco-efficiency; it comprehensively evaluated MSWIFA’s sustainable application potential in UHPC systems to provide scientific support for reducing energy consumption and CO_2_ emissions while enabling efficient resource recovery and stabilization of hazardous wastes like MSWIFA, demonstrating substantial environmental and economic benefits.

## 2. Materials and Methods

### 2.1. Raw Materials

The cement employed was P·O 52.5, with its fundamental properties detailed in Table 1. The ternary cementitious system, comprising cement, slag and silica fume, significantly enhancing the compactness of the paste. Silica fume has a specific surface area of 1800 m^2^/kg, an apparent density of 2.20 g/cm^3^, and a moisture content of 0.05%. The slag exhibits a specific surface area of 430 m^2^/kg, a density of 3.10 g/cm^3^, and a moisture content of 0.43%. MSWIFA, provided by an environmental energy company in Henan Province, partially replaced cement. The quartz sand (QS) had a particle size range of 0.15–1.18 mm, of which the mass ratio of 0.15–0.30 mm, 0.30–0.60 mm and 0.60–1.18 mm is 2:4.2:3.8. The chemical compositions of raw materials are presented in Table 2. The polycarboxylate superplasticizer is a pale yellow viscous liquid with a water reduction rate of ≥ 40% and a solid content of 18%.

**Figure 1:**
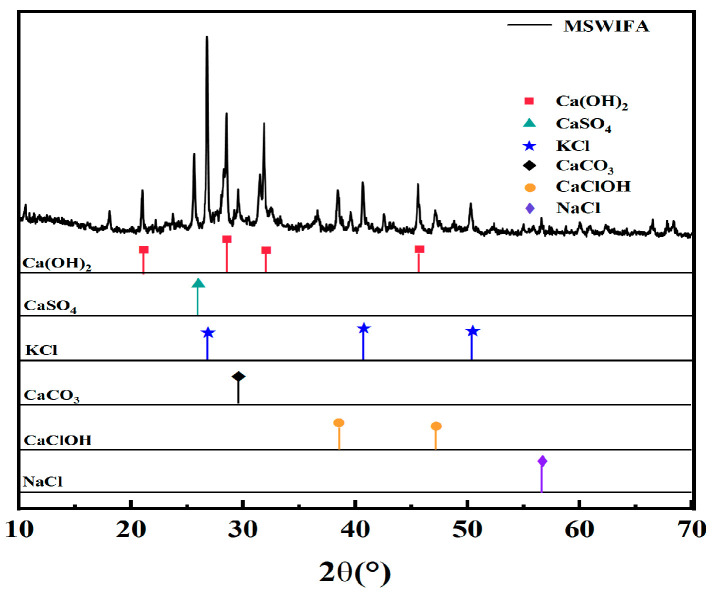
XRD pattern of MSWIFA.

**Figure 2:**
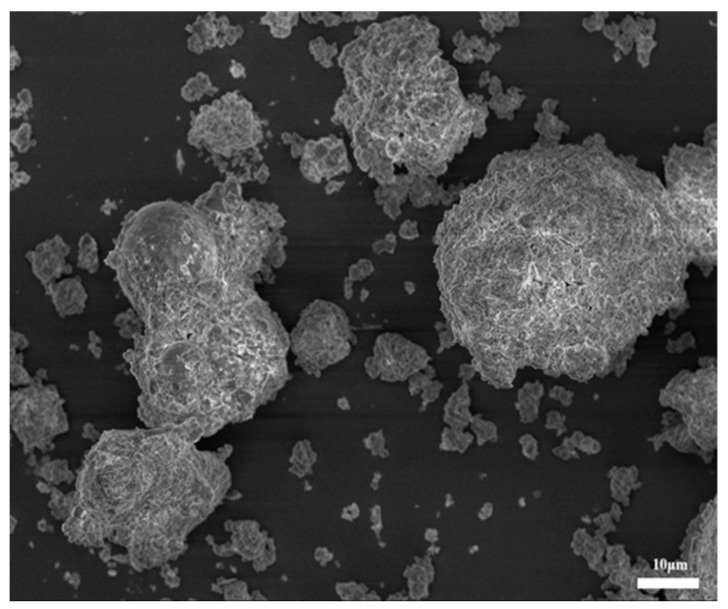
SEM image of MSWIFA.

As shown in Table 2, MSWIFA exhibits high CaO content reaching 43.06%, similar to cement clinker composition. This enables it to function as a supplementary cementitious material through hydration reactions forming calcium silicate hydrate (C-S-H) [10]. According to EN 206:2013+A2:2021, the water-soluble chloride content in concrete must not exceed 0.10% of the cement mass, so the MSWIFA dosage must be strictly controlled to meet this requirement. The mineral composition and microscopic morphology of MSWIFA are presented in Figure 1 and Figure 2, respectively. Figure 1 reveals a prominent and sharp KCl diffraction peak at approximately 27°, indicating good crystallinity. Concurrently, numerous wavy amorphous peaks in the diffraction pattern demonstrate the presence of substantial glassy amorphous phases in MSWIFA [36]. Furthermore, heavy metals in MSWIFA exist in compound forms and primarily reside within amorphous phases.

Figure 2 shows MSWIFA particles exhibiting flocculent, flaky, platy, and irregular morphologies. Numerous layered flaky crystals are loosely arranged with uneven surfaces, resulting in a large specific surface area.

**Figure 3:**
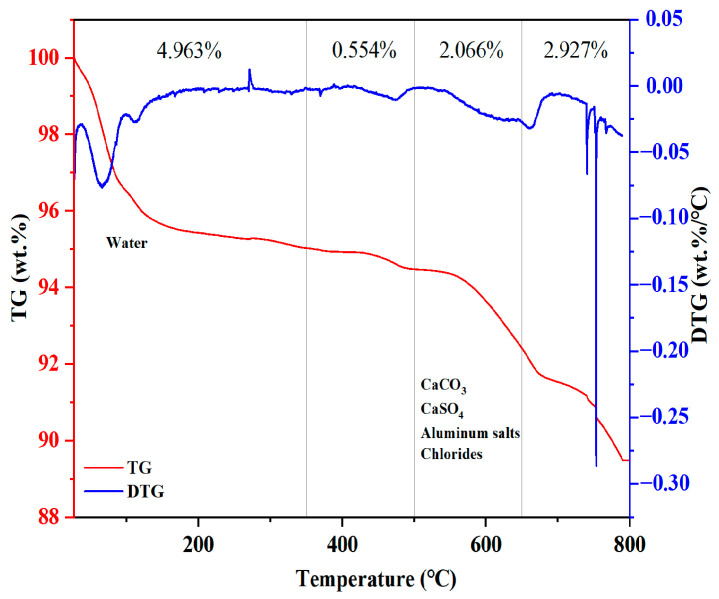
TG and DTG analysis of MSWIFA.

The TG/DTA curve of MSWIFA is shown in Figure 3. As shown in the figure, desorption of physically adsorbed water occurs primarily within the 100–350 °C range. Between 350 and 500 °C, mass loss corresponds to evaporation of free and bound water along with oxidation and decomposition of organic matter. The mass reduction observed between 50 and 500 °C originates from thermal decomposition of calcium carbonate, calcium sulfate, aluminum salts, and chlorides. As the temperature increased, MSWIFA exhibited gradual mass loss between 0 and 800 °C, with a total mass loss of 10.51%. This suggests that MSWIFA has good thermal stability during the curing and service stages of UHPC.

**Figure 4:**
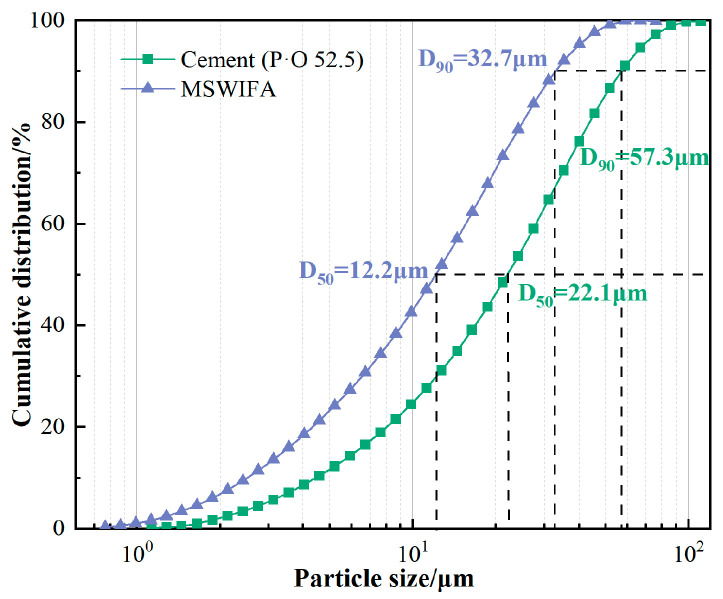
Particle size of cement and MSWIFA.

The particle size distribution characteristics of MSWIFA are shown in Figure 4. The particle size distribution parameters indicate that the median particle size (D50) is 12.2 μm, and the D90 control particle size is 32.7 μm. Compared to cement, the D50 (22.1 μm) and D90 (57.3 μm) are reduced by 44.8% and 42.9%, respectively. This ultra-fine micron-scale particle size distribution endows MSWIFA with physical filling effects and volcanic ash effect activation in the UHPC system, demonstrating its potential as a low-carbon alternative.

The heavy metal leaching test data for MSWIFA are presented in Table 3. As shown in the table, the leaching concentrations of Pb and Cd in MSWIFA are 5.18 mg/L and 1.83 mg/L, respectively, both of which exceed the standard limits for leaching toxicity of hazardous waste in China, the Chinese landfill control standards, and the EU standard limits for leaching toxicity of hazardous waste. In addition, the leaching concentration of Cr (0.72 mg/L) also exceeds the Chinese landfill control standards.

### 2.2. Preparation of UHPC Samples

Based on the optimized mix proportion established in preliminary trials as the reference group (Ref group), this study prepared MSWIFA-UHPC specimens by replacing cementitious materials with MSWIFA at 6%, 12%, 18%, and 24% substitution rates. The corresponding sample numbers were: MSWIFA-6, MSWIFA-12, MSWIFA-18, MSWIFA-24. All replacement groups maintained the same water-to-binder ratio and cementitious material content as Ref group, with only alterations to the binder composition. Detailed experimental mix proportion are shown in Table 4.

The specimen preparation procedure is as follows: first, water and superplasticizer were uniformly mixed to form a solution. Cement, silica fume, slag, and MSWIFA were then poured into a mortar mixer and mixed at low speed for 60 s. Subsequently, 80% of the superplasticizer solution was added and mixed at low speed for 120 s. QS was then incorporated with continued low speed mixing for 120 s. Finally, the remaining 20% superplasticizer solution was added to the mixture and mixed at high speed for 360 s. The mixed slurry was poured into half-filled 40 mm × 40 mm × 160 mm molds and vibrated for 60 s, after which the molds were completely filled and vibrated for an additional 60 s. Surfaces were scraped smooth and covered with plastic wrap, and transferred to a standard curing chamber for 24 h before demolding, and specimens were then standard-cured until designated ages for performance testing.

### 2.3. Experimental Methods

#### 2.3.1. Physical and Mechanical Properties

**Figure 5:**
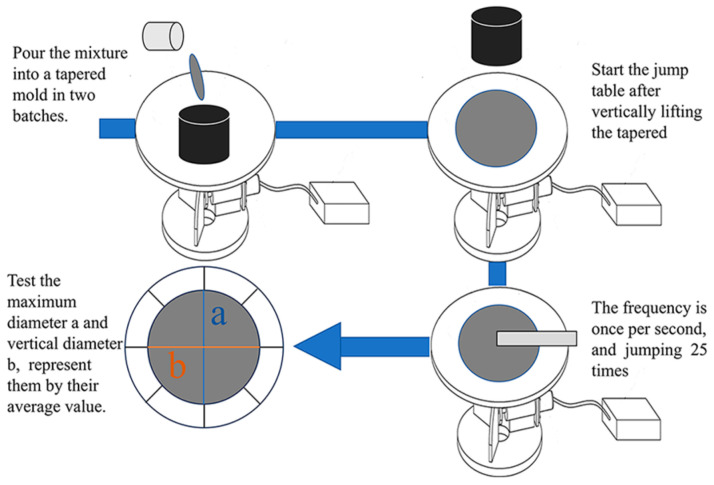
Schematic diagram of the mixture flow test process.

To test the fluidity of the specimens according to the GB/T 2419-2005 [37], the fluidity of the specimen was determined using the jump table method, a and b denote the maximum diameter of the expanded base and its perpendicular diameter respectively. The specific testing procedure is shown in Figure 5.

According to the national standard JGJ/T 70-2009 [38], the initial setting time and final setting time were determined using the standard penetration resistance method. The initial setting time was recorded when the penetration resistance reaches 0.3 MPa, and the final setting time was recorded when the resistance value reaches 0.7 MPa.

The effect of MSWIFA replacement rate on the wet packing density of UHPC slurry was determined [39]. The calculation method for the wet packing density of UHPC is shown in Equation (1):

(1) $$\Phi \;=\;\frac{V_{s}}{V},$$

where Φ is the packing density, V is the volume of the mold to be filled by the sample, the container volume used in this paper is 400 mL, and Vs is the total volume of concrete raw materials. Vs can be expressed as Equation (2):

(2) $$V_{s}\;=\;\frac{M}{\rho_{w}R_{w}+\rho_{a}R_{a}+\sum_{x\in ∁}\;\rho_{x}R_{x}},$$

where M is total amount of concrete mixture in the container; x represents different cementitious materials; ∁ represents all cementitious material components; $\rho_{w},\;\;\rho_{a}\;\mathrm{and}\;\rho_{x}$ represent densities of water, aggregate (QS) and cementitious material x respectively; $R_{w},\;R_{a}\;\mathrm{and}\;R_{x}$ respectively represent the volumetric ratios of water, aggregate and cementitious materials x to the total volume of concrete raw materials.

According to the GB/T17671-2021 [40], the mechanical properties of UHPC were tested. The sample size was 40 mm × 40 mm × 160 mm, and the compressive and flexural strengths of UHPC at 3, 7, and 28 days were tested. Tests were performed on a WDW-600E electro-hydraulic universal testing machine at loading rates of 0.05 kN/s for flexure and 2.4 kN/s for compression.

#### 2.3.2. Durability

According to the GB/T 50082-2024 [41], the specimen dimensions were 25 mm × 25 mm × 280 mm. After 24 h of initial curing, the demolded specimens were oriented and marked, then subjected to 48 h of water immersion to stabilize the dimensional baseline. Shrinkage over time was analyzed through regular dimensional monitoring, and deformation values were calculated using the formula in Equation (3), with the initial length denoted as L_0_ and the final length recorded as L_t_.

(3) $$\varepsilon_{t}=\frac{L_{0}-L_{t}}{L_{b}},$$

$\varepsilon_{t}$: Dry shrinkage of specimen at t-day (10^−6^); $L_{0}$: Initial length of specimen (mm); $L_{t}$: T-day length of specimen (mm); $L_{b}$: Gauge length (mm).

To test the resistance of UHPC to chloride and sulfate attack, specimens cured for 28 days were immersed in a 10% Na_2_SO_4_ solution and a 5% NaCl solution by mass, respectively. After soaking for 28 and 56 days, the specimens were removed, wiped dry, and their mass and mechanical strength were measured. The mass loss rate and corrosion resistance coefficient were calculated using Equations (4) and (5), respectively.

(4) $${\Delta W}_{n}=\frac{W_{0}-{\;W}_{n}}{W_{0}}\times 100\%,$$

(5) $$K_{f}=\frac{f_{i}}{f_{0}}\times 100\%,$$

${\Delta W}_{n}$: Mass loss rate after chloride or sulfate attack at the corresponding age (%); $W_{0}$: Mass after water immersion at the corresponding age (g);$\;W_{n}$: Mass of specimen after chloride or sulfate attack at the corresponding age (g); $K_{f}$: Compressive strength corrosion resistance coefficient at the corresponding age (%); $f_{i}$: Compressive strength of specimens after chloride or sulfate attack at the corresponding age (MPa); $f_{0}$: Compressive strength of specimens after water immersion at the corresponding age (MPa).

#### 2.3.3. Microscopic Properties

Chemical composition analysis was performed using an S8 TIGER wavelength dispersive X-ray fluorescence spectrometer. X-ray diffraction (XRD) analysis was performed using the D8-ADVANCE X-ray diffractometer, with a scanning step of 0.02°, a scanning range of 10–80°, and a scanning speed was 10°/min. Thermogravimetric analysis (TGA) was performed using a TGA 400 synchronous thermal analyzer under a nitrogen atmosphere, with a heating rate of 10 °C/min and a temperature measurement range of 30–800 °C.

Samples were cut into 5 mm × 5 mm × 5 mm cubic blocks from the middle of 28-day specimens, ground with sandpaper, immersed in anhydrous ethanol to terminate hydration, then dried at 40 °C to constant weight. After gold spraying, testing was conducted using a Geminisem scanning electron microscope in secondary electron imaging mode.

#### 2.3.4. Heavy Metal Leaching Concentration Analysis

Tests were conducted in accordance with the national environmental protection industry standards HJ/T 300-2007 [42] and HJ 557-2010 [43]. The test samples were crushed and placed in centrifuge tubes for rotational oscillation treatment. The mixture was then filtered using a microporous membrane, and the filtrate was analyzed using inductively coupled plasma mass spectrometry (ICP-MS) to determine the concentration of heavy metals.

## 3. Results and Discussion

### 3.1. Effects of MSWIFA Content on the Physical Properties of UHPC

#### 3.1.1. Fluidity

**Figure 6:**
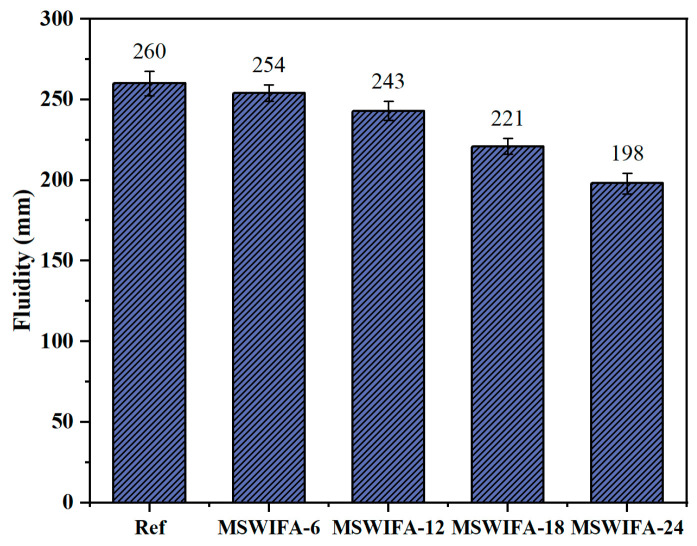
Fluidity of UHPC at different MSWIFA replacement rates.

The results of the UHPC fluidity test at different MSWIFA replacement rates are shown in Figure 6. The fluidity of the reference group was 260 mm, and the fluidity of UHPC gradually decreased with increasing MSWIFA replacement rate. When the MSWIFA replacement rates were 6%, 12%, 18%, and 24%, respectively, the UHPC fluidity decreased by 2.31%, 6.54%, 15.01%, and 23.85% compared to the reference group. Notably, even at the maximum replacement rate of 24%, the fluidity still reached 198 mm, meeting the workability requirements for UHPC. This phenomenon primarily stemmed from two material characteristics of MSWIFA. First, its porous morphology and substantially larger specific surface area compared to cement increased free water adsorption per unit mass, thereby effectively reducing the water-to-binder ratio and elevating paste viscosity. Second, it exhibited irregular angular geometries rather than the spherical morphology characteristic of cement grains, significantly amplified interparticle friction. More significantly, the larger specific surface area enhanced preferential adsorption of water reducing agents. As the replacement rate increased, more agents were adsorbed onto MSWIFA particles, weakening the lubricating effect on cement particles and thereby preventing full utilization of water-reducing properties. On the other hand, alkaline components (e.g., Na_2_O, K_2_O) in MSWIFA might interact with other materials, altering hydration kinetics and consequently affecting paste viscosity. In conclusion, when the proportion of MSWIFA replacing cement did not exceed 24%, the fluidity of UHPC met the requirements.

#### 3.1.2. Setting Time

**Figure 7:**
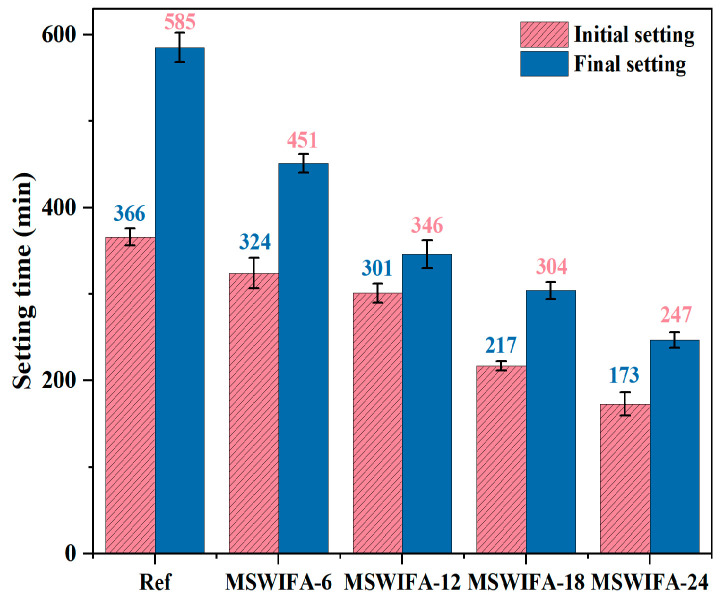
Setting time of UHPC at different MSWIFA replacement rates.

The test results for the setting time of UHPC under different MSWIFA replacement rates are shown in Figure 7. From the test results, it can be observed that as the MSWIFA replacement rate increases, both the initial setting time and final setting time show a significant decreasing trend. When the MSWIFA replacement rates for cement are 6%, 12%, 18%, and 24%, the initial setting times decrease by 22.91%, 40.85%, 48.03%, and 57.78%, respectively; the final setting time decreased by 11.48%, 17.76%, 40.71%, and 52.73%, respectively, compared with the reference group.

This phenomenon was primarily attributed to alkaline components in MSWIFA, such as Na_2_O and K_2_O, which reacted with gypsum (CaSO_4_·2H_2_O) in cement to form crystalline products like Na_2_SO_4_, thereby accelerating cement hydration and reducing the setting time of UHPC. Additionally, Cl^−^ and heavy metal ions (Pb^2+^, Cd^2+^, Zn^2+^, etc.) in MSWIFA significantly shortened the initial and final setting times. The small ionic radius of Cl^−^ enabled its penetration through the hydration product shells of C_3_S/C_2_S enter the pore solution, disrupted local charge balance, hindered OH^−^ reverse diffusion, and caused Ca^2+^ to precipitate rapidly as Ca(OH)_2_ crystals, thereby accelerating the hydration process and shortening the setting time. Furthermore, Cl^−^ reacted with C_3_A to form Friedel’s salt (C_3_A·CaCl_2_·10H_2_O). The specific reaction formulas are shown in Equations (6) and (7), respectively. The rapid consumption of Ca^2+^ during this reaction destabilized the dissolution equilibrium of C_3_S, inducing accelerated precipitation of C-S-H gel and shortened setting time reduction. Heavy metal ions in MSWIFA, such as Cd^2+^, reacted with OH^−^ to form insoluble Cd(OH)_2_ precipitates, which caused the cement paste to harden, thereby accelerating the setting of UHPC; Zn^2+^ formed Zn(OH)_2_ colloids and reacted with CO_3_^2−^ to form CaCO_3_, both of which filled pores and formed a rigid framework, thereby shortening the setting time; Cr^3+^ preferentially adsorbed onto surface defects of C_3_S during the early stages of hydration, increased the rate of C-S-H formation, accelerated structural densification, thereby accelerating the setting of UHPC.

C_3_A + Ca(OH)_2_ + 11 H_2_O → C_4_AH_13_ (6)

C_4_AH_13_ + 2 Cl^−^ → C_3_A·CaCl_2_·10H_2_O + OH^−^ (7)

In summary, the addition of MSWIFA significantly affected the setting time of UHPC. As the replacement rate of MSWIFA increased, both the initial and final setting times of UHPC decreased, indicating that MSWIFA optimized the setting performance of cement to some extent. However, MSWIFA had low pozzolanic activity, and its promotional effect on cement hydration reactions was limited. Therefore, at high replacement rates, the compressive strength of UHPC may have decreased. Overall, the addition of MSWIFA not only reduced the setting time of UHPC but also optimized the material’s pore structure and durability to some extent, providing new insights for the sustainable development of UHPC.

#### 3.1.3. Wet Packing Density

**Figure 8:**
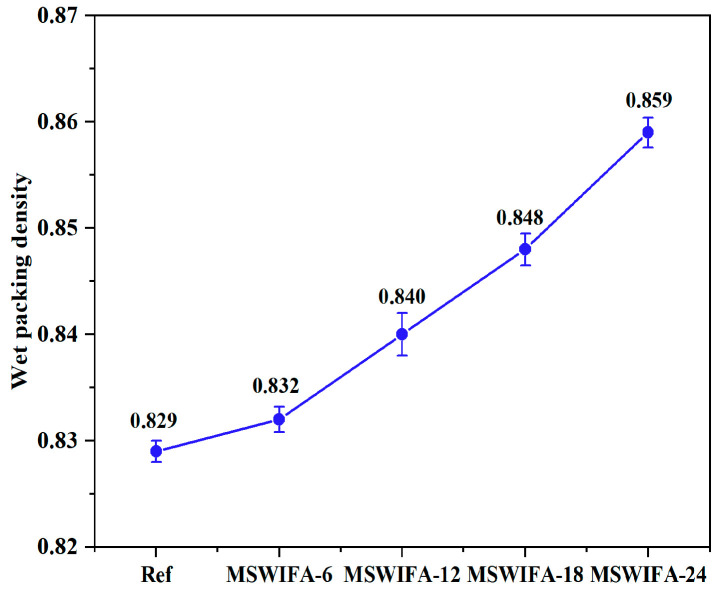
Wet packing density of UHPC at different MSWIFA replacement rates.

The wet packing density test results of UHPC under different MSWIFA replacement ratios are presented in Figure 8. As illustrated, substituting cement with MSWIFA enhanced the wet packing density of UHPC. Compared to the reference group, the MSWIFA-6, MSWIFA-12, MSWIFA-18, and MSWIFA-24 groups exhibited density increases of 0.32%, 1.33%, 2.29%, and 3.62%, respectively, indicating statistically significant yet gradual improvements. Additionally, the surface roughness of MSWIFA was significantly higher than that of cement, which promoted the penetration of water molecules into pores via capillary action in a wet state, forming a gel-like layer that reduced interparticle friction, facilitated particle reorganization and compaction, thereby decreasing pore connectivity in the cement paste and increasing wet packing density. Simultaneously, in the alkaline environment, MSWIFA released reactive SiO_2_/Al_2_O_3_, which reacted with Ca(OH)_2_ from cement hydration to form C-S-H gels coating particle surfaces. This reaction mitigated defects in the interfacial transition zone (ITZ) and enhanced interparticle bonding efficiency. Although MSWIFA incorporation significantly improved UHPC’s wet packing density, its enhancement of mechanical properties was limited. At higher replacement levels, mechanical performance was even compromised. This limitation was attributed to the inherently low pozzolanic reactivity of MSWIFA (restricting its ability to accelerate cement hydration) and the reduced space for hydration products due to dense packing, which collectively inhibited the hydration degree. Consequently, UHPC’s mechanical properties declined at elevated replacement rates. In summary, while MSWIFA addition improved material compactness and pore structure, its contribution to mechanical performance remained constrained. Further optimization of dosage is essential to balance performance benefits with environmental advantages.

### 3.2. Effects of MSWIFA Content on the Mechanical Properties of UHPC

#### 3.2.1. Compressive Strength

**Figure 9:**
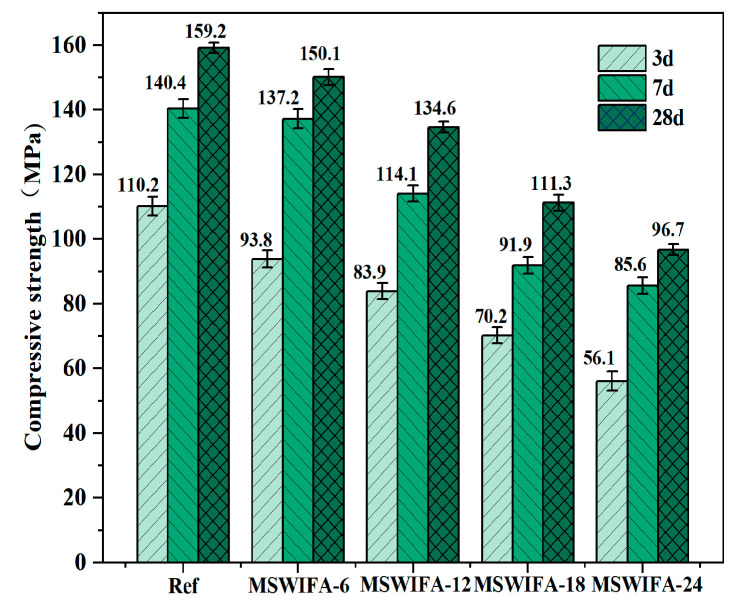
Compressive strength development of UHPC samples.

The influence of different MSWIFA replacement ratios on the compressive strength of UHPC was presented in Figure 9. The compressive strength of UHPC at all curing ages demonstrated a declining trend with increasing MSWIFA replacement. The reference group (0% MSWIFA) exhibited compressive strengths of 110.2 MPa, 140.4 MPa, and 159.2 MPa at 3, 7, and 28 days, respectively. At a 6% MSWIFA replacement, the 3, 7, and 28-day compressive strengths decreased to 93.8 MPa, 137.2 MPa, and 150.1 MPa (a 5.68% reduction). At this point, the strength loss was primarily attributed to a slight reduction in the content of cement clinker (C_3_S/C_2_S). MSWIFA primarily adjusted compound content within the reaction system and improved particle size distribution, exerting minimal influence on hydration products. This micro-aggregate effect partially compensated for the loss of cementitious activity, resulting in a relatively controlled strength reduction. As the MSWIFA content further increased, compressive strength continued to decline. At a 12% MSWIFA replacement, the 28-day compressive strength was 134.6 MPa, which still met the requirements for UHPC. However, when the replacement ratio increased to 18% and 24%, the 28-day compressive strengths dropped to 111.3 MPa and 96.7 MPa, representing reductions of 30.09% and 39.24% compared to the reference group, respectively. This indicated that strength loss was relatively minor at lower replacement levels (≤12%), while a more significant decline occurred at higher dosages. Notably, at the 24% replacement level, although the compressive strength was lower than the reference group, it still satisfied the compressive strength standard for high-performance concrete (HPC).

Compared to cement, MSWIFA contained higher levels of unburned carbon and harmful substances, particularly heavy metal ions such as Pb^2+^/Cd^2+^. These ions could occupy binding sites in C-S-H gels while inhibiting cement hydration reactions through adsorption of Ca^2+^ or formation of complex species, ultimately compromising the mechanical performance of the final product. The pozzolanic activity of MSWIFA was significantly lower than that of cement. When the replacement ratio exceeded 6%, the reduced cement content resulted in insufficient production of hydration products (C-S-H gels), leading to diminished strength in UHPC. Additionally, replacing cement with MSWIFA altered UHPC’s microstructure. MSWIFA’s irregular particle morphology and porous features weakened the ITZ and degraded pore structure. Excessive pores reduced UHPC’s integrity and compressive strength. Additionally, MSWIFA’s distinct alkali composition disrupted the system’s alkali equilibrium, potentially causing expansion and matrix damage. This imbalance further affected UHPC’s strength development.

#### 3.2.2. Flexural Strength

**Figure 10:**
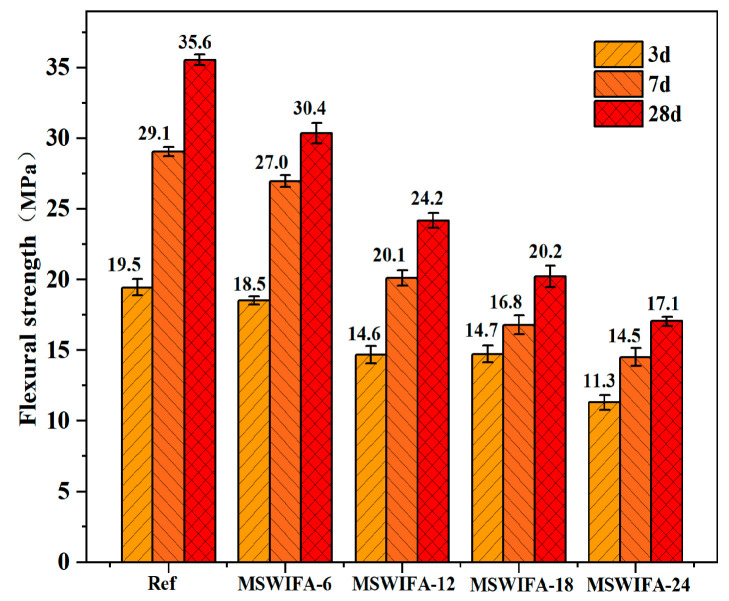
Flexural strength development of UHPC samples.

The influence of different MSWIFA replacement ratios on UHPC flexural strength was presented in Figure 10. As shown, at different MSWIFA content levels, the flexural strength of UHPC at all ages was lower than that of the reference group. The flexural strength of UHPC decreased with increasing MSWIFA replacement rate, and this trend was consistent with that of compressive strength. The reference group (0% MSWIFA) had a flexural strength of 35.6 MPa at 28 days. When the MSWIFA replacement rate was increased to 6%, 12%, 18%, and 24%, the flexural strength at 28 days decreased by 14.57%, 31.41%, 43.11%, and 52.02%, respectively. In UHPC, the ITZ between aggregates and cementitious materials played a crucial role in concrete strength. When MSWIFA replaced cement, the wet packing density continued to increase. However, MSWIFA’s large specific surface area adsorbed water and mortar residues, potentially leading to weakened bond strength at the cement–aggregate interface and promoting a porous ITZ structure that compromised UHPC strength. Additionally, MSWIFA contained elevated K_2_O and Na_2_O, the competitive adsorption of Na^+^/K^+^ on the C-S-H gel surface inhibited the growth of hydration products and further reduced the flexural strength of UHPC.

### 3.3. Effects of MSWIFA Content on the Durability of UHPC

#### 3.3.1. Drying Shrinkage Performance

**Figure 11:**
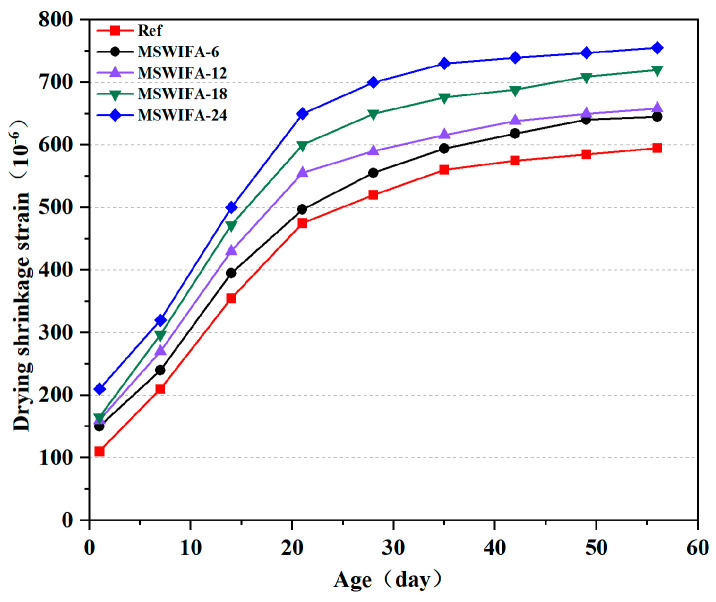
Drying shrinkage performance of UHPC samples.

The drying shrinkage test results of UHPC with different MSWIFA replacement ratios are illustrated in Figure 11. The dry shrinkage rate of the reference group UHPC at 7 days was 210 × 10^−6^, and it increased to 520 × 10^−6^ at 28 days, with the 28-day dry shrinkage rate accounting for 87.39% of the 56-day value. The reference group specimens exhibited relatively high dry shrinkage rates, attributed to the stronger chemical shrinkage effect caused by the pozzolanic reaction of slag compared to cement hydration, which accelerated internal water consumption. Concurrently, the secondary reaction of highly active SiO_2_ in silica fume with Ca(OH)_2_ further promoted hydration process, with both factors jointly exacerbated the self-contraction effect.

As shown in the figure, the shrinkage behavior of UHPC followed a consistent pattern. As age increased, the shrinkage rate of UHPC gradually rose, and the growth rate slowed after 28 days. During the initial 28 days, intensive hydration reactions consumed a large amount of moisture in the low water-to-binder ratio system. Intensive hydration reactions consumed a large amount of moisture, leading to rapid water loss in the micro-capillaries and rapid shrinkage development. After 28 days, the accumulation of hydration products reduced capillary pressure, causing water molecules to migrate more slowly, thereby reducing the rate of drying shrinkage development compared with that before 28 days. When the MSWIFA replacement rate was set at 6%, 12%, 18%, and 24%, the 28-day drying shrinkage rates of the specimens increased by 6.73%, 13.46%, 25%, and 25.71%, respectively, compared to the reference group. The maximum increase in drying shrinkage rate occurred at the 24% replacement level. This was attributed to the porous nature of MSWIFA, which accelerated water migration within the system. On the other hand, as the MSWIFA replacement rate increased, the hydration reaction of the entire cementitious system was significantly affected, resulting in a reduction in the amount of hydration products and an increase in the number of harmful pores. These discontinuous pore networks accelerated water loss and thereby increased the drying shrinkage rate. According to GB/T 50082-2024 [41], the 28-day drying shrinkage rate of UHPC was required to be controlled within 600 × 10^−6^. In this study, a UHPC mixture with an MSWIFA replacement rate of 12% was used, and its 28-day drying shrinkage rate was 576 × 10^−6^, indicating that its drying shrinkage performance satisfied the relevant standard requirements.

#### 3.3.2. Resistance to Chloride Attack

**Figure 12:**
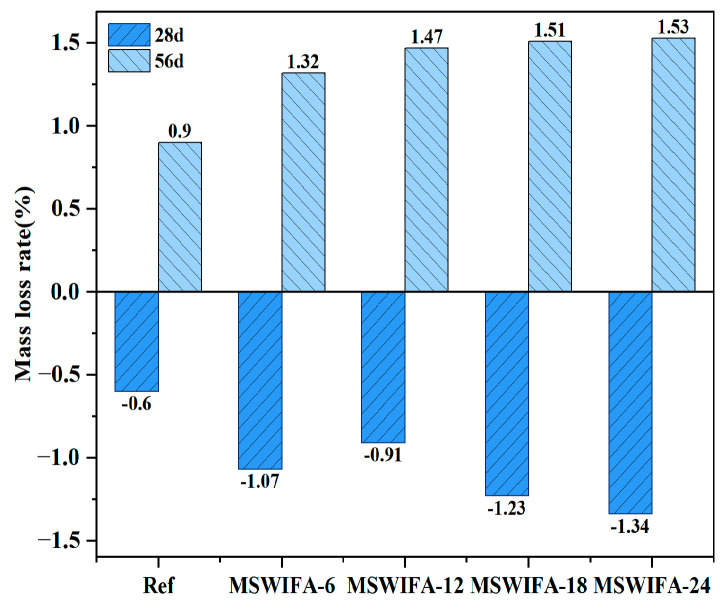
Mass loss rate of UHPC with different MSWIFA content under NaCl solution immersion.

The mass loss ratios of UHPC specimens under NaCl erosion at varying MSWIFA replacement ratios are presented in Figure 12. The mass evolution of UHPC specimens immersed in NaCl solution exhibited a distinct two-stage characteristic. After 28 days of immersion in NaCl solution, the mass of Ref, MSWIFA-6, MSWIFA-12, MSWIFA-18, and MSWIFA-24 groups increased by 0.6%, 1.07%, 0.91%, 1.23%, and 1.34%, respectively.

This phenomenon was primarily attributed to the porous structure of MSWIFA and its significant increase in capillary porosity, which enabled rapid penetration of NaCl solution into the material through capillary action, thereby accelerating Cl^−^ diffusion. Concurrently, abundant Cl^−^ in the solution chemically reacted with unhydrated aluminates in the cementitious system to form Friedel’s salt. Furthermore, the ingress of Cl^−^ into the UHPC matrix facilitated the combination with Ca^2+^, promoting the formation of soluble CaCl_2_. This crystallization process induced temporary mass gain, which may correlating with the mass loss observed in TGA curves at 650–750 °C due to chloride decomposition. However, under prolonged immersion in NaCl solution, mass loss became the dominant phenomenon.

At 56 days of curing, UHPC exhibited positive mass loss ratios that increased with higher MSWIFA replacement rates. The mass losses for each group measured 0.9%, 1.32%, 1.47%, 1.51%, and 1.53%, respectively. This transition resulted from the formation of expansive corrosion products such as Friedel’s salt and CaCl_2_ crystals under sustained chloride attack. Accumulation of these products generated internal expansive stresses, initiating microcracks on the UHPC surface and even causing material spalling, ultimately leading to mass loss. Consequently, elevated MSWIFA replacement ratios accelerated corrosion rates and increased internal porosity in UHPC.

**Figure 13:**
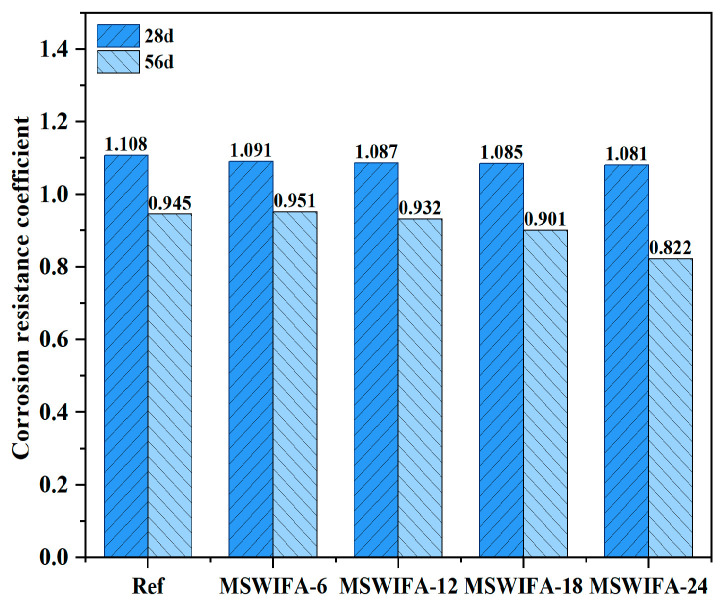
Corrosion resistance coefficient of UHPC with different MSWIFA content under NaCl solution immersion.

The test results for the corrosion resistance coefficient of UHPC under different MSWIFA replacement rates are shown in Figure 13. As shown in the figure, the compressive strength of UHPC specimens immersed in NaCl solution initially increased but subsequently decreased with prolonged curing duration. After 28 days of NaCl exposure, the corrosion resistance coefficient exceeded 1, indicating that the compressive strength of UHPC with different replacement ratios surpassed that of the samples immersed in clean water. However, as the curing time extended to 56 days, the corrosion resistance coefficient dropped below 1, signifying varying degrees of compressive strength reduction and reflecting a gradual decline in Cl^−^ erosion resistance. The corrosion resistance coefficient at a replacement rate of 6% was greater than that of the reference group with a replacement rate of 0%. However, when the MSWIFA replacement rate exceeded 6%, the corrosion resistance coefficient gradually decreased.

The increase in compressive strength at 28 days was attributed to the continued hydration of unreacted cementitious materials within the specimens during the early stages of the test, producing hydration products such as C-S-H (from the hydration of C_3_S) that filled the micro-pores in the UHPC, resulting in a denser matrix. On the other hand, a small amount of corrosion product, Friedel’s salt, generated under chloride salt erosion, also filled the internal pores of the UHPC, contributing to the improvement in compressive strength during the initial erosion period. However, after 56 days of immersion in NaCl solution, strength loss became the dominant phenomenon. This transition was primarily due to the accumulation of Friedel’s salt to a critical level under prolonged NaCl solution erosion, generating expansive stress, inducing microcracks on the UHPC surface and even caused material spalling. Additionally, when UHPC was exposed to NaCl solution for an extended period, Cl^−^ in the solution reacted chemically with Friedel’s salt, potentially leading to its decomposition or dissolution into the solution, which resulted in a decrease in the compressive strength of UHPC.

At a replacement rate of 6%, MSWIFA introduced an appropriate amount of internal porosity into UHPC. Compared with the control group, MSWIFA-6 exhibited a higher corrosion resistance coefficient and effectively reduced expansion stress. However, when the replacement rate exceeded 6%, the increased MSWIFA content led to a more connected capillary pore network, significantly increasing the diffusion coefficient of Cl^−^. Notably, even at a replacement rate of 12%, the compressive strength of UHPC remained above 120 MPa after 56 days of immersion in a NaCl solution, meeting the mechanical performance requirements for UHPC.

#### 3.3.3. Resistance to Sulfate Attack

**Figure 14:**
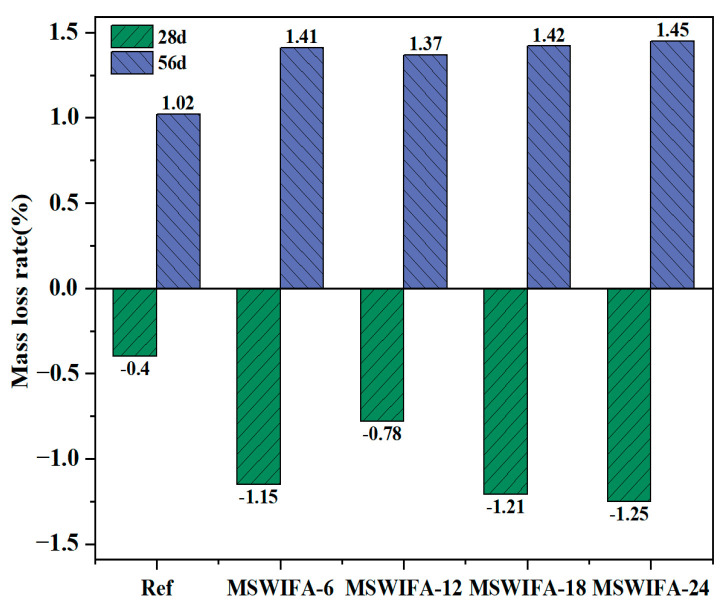
Mass loss rate of UHPC with different MSWIFA content under Na_2_SO_4_ solution immersion.

The mass loss rate of UHPC immersed in Na_2_SO_4_ solution is shown in Figure 14. Under sulfate attack, the sulfate resistance of UHPC exhibited a non-linear relationship with the municipal solid waste incineration fly ash (MSWIFA) replacement ratio. The study found that the local Al/Ca ratio gradient caused by the uneven dispersion of MSWIFA was the primary cause of mass change. As shown in the figure, at the 28-day curing age, the Ref, MSWIFA-6, MSWIFA-12, MSWIFA-18, and MSWIFA-24 samples increased by 0.40%, 1.15%, 0.78%, 1.21%, and 1.25%, respectively, compared to samples immersed in clean water. This mass gain was attributed to the porous nature of MSWIFA, which significantly enhanced the capillary water absorption capacity of UHPC, facilitating greater ingress of water and Na_2_SO_4_ solution into the matrix. Concurrently, the infiltrated SO_4_^2−^ reacted with unhydrated aluminates such as C_3_A to form expansive products, including ettringite (AFt) and CaSO_4_·2H_2_O, leading to short-term mass increase. At the 56-day curing age, the masses of the Ref, MSWIFA-6, MSWIFA-12, MSWIFA-18, and MSWIFA-24 samples after immersion in Na_2_SO_4_ solution decreased by 1.02%, 1.41%, 1.37%, 1.42%, and 1.45%, respectively, compared to the clean water group samples. This mass loss was due to microcrack propagation initiated by expansion stress from excessive AFt accumulation, allowing leaching of material. Additionally, under prolonged exposure, the low-calcium environment and high SO_4_^2−^ concentration promoted the decomposition of AFt into soluble Al(OH)_3_ gel, which was leached out, leading to mass loss.

The non-linear evolution of UHPC quality under sulfate attack was fundamentally attributed to the combined effect of a critical decrease in pore solution Ca^2+^ concentration and local chemical heterogeneity. As unhydrated C_3_S/C_2_S continuously consumed Ca^2+^ to generate C-S-H gel, while SO_4_^2−^ combined with Ca^2+^ to form gypsum precipitate. As sulfate concentration declined, the thermodynamic stability of AFt decreased, triggering its conversion to AFm, while the uneven distribution of MSWIFA induced micro-scale Al/Ca ratio differences—promoting the preferential transformation of AFt into aluminum-rich AFm (characterized by low shrinkage but prone to hydrolysis), whereas in regions with low Al/Ca ratio, it decomposed into soluble Al(OH)_3_ and CaSO_4_·2H_2_O. This phase transformation pathway branching, driven by local chemical gradients rather than a single substitution rate variable, determined the non-linear degradation behavior.

**Figure 15:**
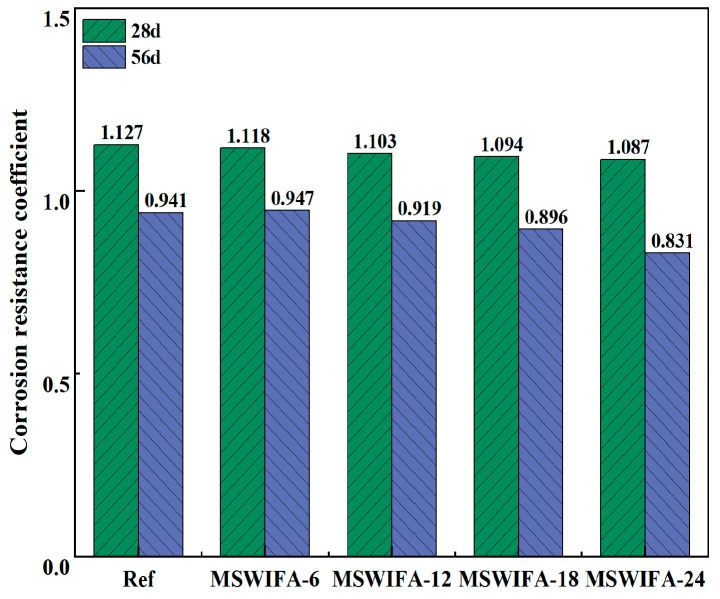
Corrosion resistance coefficient of UHPC with different MSWIFA content under Na_2_SO_4_ solution immersion.

The corrosion resistance coefficient test of UHPC after immersion in Na_2_SO_4_ solution is shown in Figure 15. As shown in the figure, after 28 days of Na_2_SO_4_ attack, the corrosion resistance coefficients of Ref, MSWIFA-6, MSWIFA-12, MSWIFA-18, and MSWIFA-24 were 1.127, 1.118, 1.103, 1.094, and 1.087, respectively, indicating that the compressive strength of UHPC with different substitution rates was higher than that of the samples in clean water. However, by 56 days of Na_2_SO_4_ attack, the corrosion resistance coefficients for Ref, MSWIFA-6, MSWIFA-12, MSWIFA-18, and MSWIFA-24 were 0.941, 0.947, 0.919, 0.896, and 0.831, respectively, all coefficients fell below 1.0, higher MSWIFA replacement correlated with greater strength loss, indicating reduced sulfate resistance.

The porous structure of MSWIFA significantly increased pore volume and promoted permeability of UHPC, facilitating the accelerated penetration of Na_2_SO_4_ solution into the matrix interior. The infiltrated SO_4_^2−^ reacted with unhydrated aluminate phases such as C_3_A to generate expansive products including AFt and CaSO_4_·2H_2_O. These products filled micron-sized pores and optimized pore connectivity, thereby improving the material compactness in the short term and enhancing mechanical properties. However, with prolonged curing time, the continuous generation of excessive AFt and CaSO_4_·2H_2_O induced the accumulation of internal expansive stress, triggering the propagation of microcracks along the ITZ. Simultaneously, the unhydrated C_3_S/C_2_S continuously consumed Ca^2+^, and SO_4_^2−^ combined with Ca^2+^ to form gypsum precipitation, leading to a decrease in Ca^2+^ concentration in the pore solution to a critical level. This promoted the destabilization and decomposition of AFt into soluble Al(OH)_3_ gel and loose gypsum crystals. Furthermore, the micro Al/Ca ratio gradient caused by the uneven dispersion of MSWIFA particles further bifurcated the phase transformation pathway. In high-Al regions, low-stability aluminum-rich AFm phases were generated, while in low-Al regions, soluble Al(OH)_3_ was formed. The resulting synergistic effect of non-uniform expansion and dissolution ultimately manifested as a non-linear change in the compressive strength decay rate. As the MSWIFA replacement rate increased, the corrosion resistance coefficient of UHPC at 56 days of curing maintained a stable level of approximately 0.90, with minimal variation compared to the reference group with 0% replacement. This indicated that, within the experimental dosage range, MSWIFA had a minor impact on the sulfate erosion resistance of UHPC. Combined with changes in mass loss rate, UHPC exhibited good resistance to SO_4_^2−^ erosion when containing MSWIFA.

### 3.4. Microscopic Test Analysis of UHPC

#### 3.4.1. XRD

**Figure 16:**
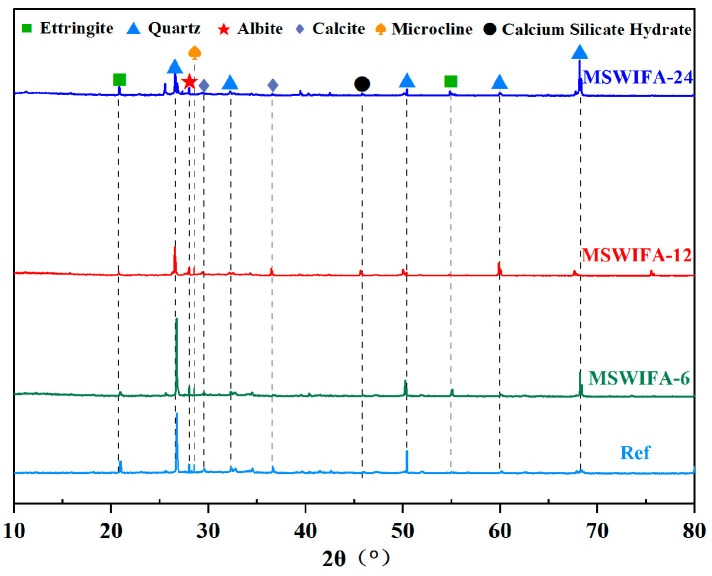
XRD diagram of UHPC with different MSWIFA content (the dashed (vertical) lines indicate the standard peak positions for each phase.).

The XRD test results of UHPC under different MSWIFA replacement rates are shown in Figure 16. As shown in the figure, the phase compositions of all specimen groups exhibited high similarity, primarily featuring characteristic peaks of mineral phases such as quartz, ettringite, albite, potassium feldspar, calcite, and calcium silicate hydrate. Quartz originated from the aggregate QS, ettringite was formed by the reaction of C_3_A in the cementitious material with sulfate, calcite was derived from the carbonation reaction of the cement hydration product Ca(OH)_2_ with CO_2_, and the formation of albite/potassium feldspar was associated with the recrystallization process of K^+^ and Na^+^ ions from MSWIFA in an alkaline solution. With the gradual increase in MSWIFA replacement ratio, the diffraction peak positions and intensities of quartz and ettringite did not show significant shifts, indicating that the introduction of MSWIFA did not cause obvious phase transformation or chemical substitution of the main crystalline phases in the matrix. However, the characteristic peak intensity of ettringite exhibited a decreasing trend. When the MSWIFA replacement ratio reached 12%, the characteristic peak of C-S-H increased. This phenomenon occurred because, with the increase in MSWIFA replacement ratio, the competitive adsorption of free water by MSWIFA particles inhibited C3A-sulfate reactions essential for ettringite formation. Furthermore, as the MSWIFA replacement ratio increased, its pozzolanic effect was enhanced, promoting further reaction of unhydrated cement particles to generate additional C-S-H gel. However, due to its low pozzolanic reactivity, the reaction rate was slow, which initially failed to fully compensate for the loss of cement hydration activity, resulting in suppressed ettringite formation. When the substitution ratio exceeded 12%, MSWIFA particles further reduced the effective water available for C_3_S hydration through competitive adsorption of free water, consequently diminishing C-S-H gel formation. This led to a reduction in the characteristic peak intensity of C-S-H gel, ultimately causing a decline in the mechanical properties of UHPC. This conclusion consisted with the compressive strength test results.

#### 3.4.2. TG/DTA

**Figure 17:**
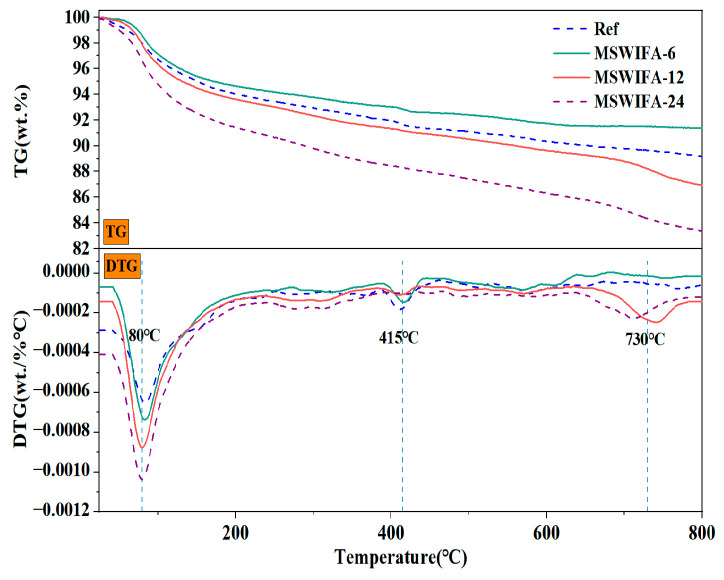
TG and DTG curves of UHPC with different MSWIFA content.

The TG and DTA curves for the Ref, MSWIFA-6, MSWIFA-12, and MSWIFA-24 specimens are shown in Figure 17. It was observed that the mass of all samples decreased with increasing temperature, which was attributed to water evaporation and decomposition of chemical components. The mass loss rates for the reference group, MSWIFA-6 group, MSWIFA-12 group, and MSWIFA-24 group were 11.97%, 8.61%, 13.07%, and 16.64%, respectively. This indicated that a 24% MSWIFA replacement ratio significantly increased the mass loss of UHPC at high temperatures, potentially due to insufficient pozzolanic activity. Under different MSWIFA replacement ratios, the overall mass loss of UHPC at elevated temperatures was relatively small, demonstrating good thermal stability.

As shown in Figure 17, the first peak occurred between 50 and 150 °C. This stage primarily associated with the loss of free water and physically adsorbed water within the UHPC, alongside the loss of chemically bound water in the C-S-H gel and AFt. Notably, the MSWIFA-24 group showed a relatively rapid mass loss rate and greater mass loss under this peak. This stemmed from MSWIFA’s higher porosity, larger specific surface area, and stronger adsorption capacity for free water. The second peak in mass loss rate occurs between 380 and 450 °C, primarily caused by the decomposition of Ca(OH)_2_. Under this characteristic peak, the reference group exhibited greater mass loss. This is because the Ca(OH)_2_ generated from the hydration of the pure cement system remains unconsumed and is entirely retained within the UHPC matrix. Additionally, as the MSWIFA replacement rate increased, the proportion of Ca(OH)_2_ consumed by pozzolanic reactions rises, leading to a decreasing mass loss. The third mass loss peak occurred at approximately 650–750 °C, resulting from the decomposition of CaCO_3_ and chlorides. Under this peak, the UHPC with MSWIFA-24 exhibited a relatively higher mass loss rate. With increasing MSWIFA replacement, the cement content decreased, reducing the total amount of hydration products (e.g., Ca(OH)_2_) and increasing pore connectivity. This promoted CO_2_ diffusion, enhanced CaCO_3_ formation, and consequently elevated the mass loss rate.

#### 3.4.3. SEM

**Figure 18:**
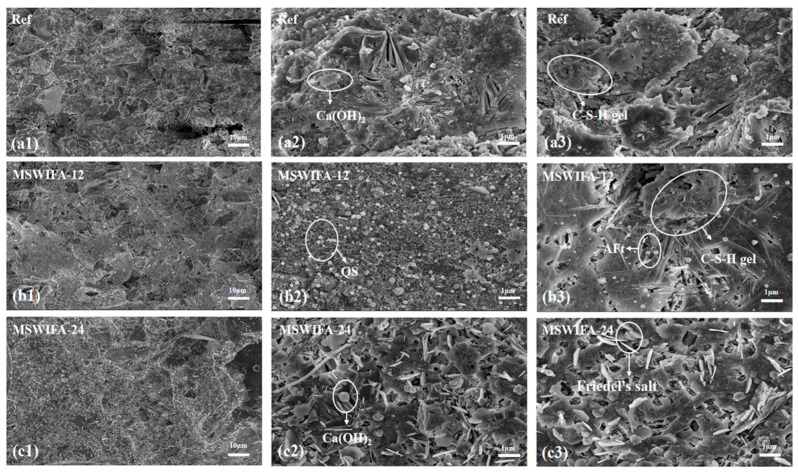
Effect of MSWIFA content on the micromorphology of UHPC: (**a1**–**a3**) reference sample; (**b1**–**b3**) 12% MSWIFA sample; (**c1**–**c3**) 24% MSWIFA sample.

The microstructural morphology test results for UHPC specimens at 28 days for the Ref, MSWIFA-12, and MSWIFA-24 groups are shown in Figure 18. In Figure 18(a3) (reference group), it can be observed that flocculent C-S-H gel as a hydration product accumulated layer by layer, while needle/rod-shaped AFt and small QS particles filled the pores. The hydration products and fine aggregates were mutually encapsulated, forming a network structure that rendered the reference group specimens dense with favorable strength development. Figure 18(b3) shows that at the 12% MSWIFA substitution rate, the overall structure of the specimens remained relatively dense; however, the formation and distribution of C-S-H gel were less uniform compared to the reference group, indicating a certain degree of structural incompleteness. As can be observed in Figure 18(c2), a significant increase in needle-shaped AFt was observed within the specimens. Concurrently, the C-S-H gel network transitioned from a flocculent structure to a more loosely flaky configuration, with visible pores appearing in some regions at the 24% MSWIFA substitution rate. Compared to the 12% MSWIFA substitution group, the 24% substitution group exhibited a marked increase in both the quantity of needle/rod-shaped AFt and the extent of the loosely flaky C-S-H gel structure.

Heavy metal ions (Zn^2+^/Pb^2+^) in MSWIFA disrupted the polymerization of C-S-H gel, transforming its morphology from a three-dimensional fibrous network into loosely stacked lamellar structures, which directly reduced gel cohesion and interfacial bonding strength. Simultaneously, higher substitution rates of MSWIFA enhanced the formation of C-A-S-H gel due to elevated aluminum and sulfur content, facilitating interlayer sliding and thereby increasing overall brittleness. Furthermore, excessive cement substitution resulted in insufficient C-S-H gel formation, leading to loosely accumulated plate-like structures with interconnected and enlarged pores. These microstructural alterations were consistent with the declining trend in mechanical properties.

### 3.5. Heavy Metal Immobilization Performance Analysis of UHPC

The ICP test data for UHPC are presented in Table 5. As shown in the table, when the replacement rate of MSWIFA was within 24%, UHPC exhibited effective solidification of heavy metals such as Pb, Cd, Cr, Zn, and Cu, with all leaching concentrations below the national safety standard limits. The efficient solidification of Pb^2+^ originated from the strong affinity of the shorter silicate tetrahedral chains in the C-S-H gel. Cd^2+^ was primarily incorporated into C-S-H through surface complexation due to charge matching constraints, while also being immobilized via physical encapsulation in pores and the formation of Cd(OH)_2_/CdCO_3_ precipitates. Cr^3+^ was effectively solidified by replacing Si^4+^ in C-S-H. Zn^2+^ formed soluble ZnCl_4_^2−^ complexes with Cl^−^ introduced by MSWIFA. Cu^2+^ was primarily immobilized through adsorption onto C-S-H gel and formation of Cu(OH)_2_ precipitates, thereby reducing mobility.

Test results indicate that when the MSWIFA replacement rate remained within 24%, the hydration products in the UHPC matrix effectively immobilized heavy metals, maintaining the leaching concentrations within safe limits and thereby meeting environmental compliance requirements. Concurrently, the compressive strength of the UHPC reached 134.63 MPa, exceeding the 120 MPa mechanical performance standard and thus meeting the strength requirements for UHPC. At MSWIFA replacement ratios below 24%, the compressive strength of UHPC was approximately 100 MPa, which fulfilled the strength requirements for HPC. Considering the balance between physical-mechanical properties and durability, UHPC with a 12% MSWIFA replacement ratio demonstrated optimal performance, establishing it as the preferred choice.

### 3.6. Integrated Ecological-Economic Analysis of UHPC

The Material Sustainability Index (MSI) serves as a parameter for evaluating a material’s environmental impact and economic performance throughout its entire lifecycle. In the application of UHPC, the MSI was employed to comprehensively assess its performance in terms of energy consumption, carbon footprint, and cost, thereby determining its sustainability. Energy consumption encompassed the energy demands during raw material processing, logistics transportation, and construction phases. Carbon footprint served as a critical indicator for quantifying greenhouse gas emissions across the entire UHPC lifecycle. Economic performance analysis was conducted based on regional market price parameters (Kaifeng City, Henan Province, China), incorporating factors such as raw material costs, production costs, transportation costs, and maintenance costs.

The unit energy consumption and CO_2_ emissions for QS [44], MSWIFA [45], cement, silica fume, slag, and water employed in the experiments were derived from literature sources [46,47]. As a product of waste incineration, MSWIFA generated virtually no additional energy consumption or carbon emissions during its application in UHPC. Energy consumption, carbon emissions, and raw material costs are presented in Table 6. Table 7 illustrates the environmental impact and economic costs associated with producing 1 kg of UHPC under varying MSWIFA substitution rates.

As shown in Table 7, the incorporation of MSWIFA significantly influenced energy consumption and carbon emissions, while its impact on raw material costs was relatively minor. This was primarily attributed to the substantial use of cement-based materials in UHPC, which resulted in high environmental energy consumption. By serving as a substitute for cement, MSWIFA maintained the ultra-high mechanical properties of UHPC while reducing the life cycle carbon footprint, thereby effectively diminishing the environmental impact of UHPC and achieving objectives of low carbon and sustainable development.

**Figure 19:**
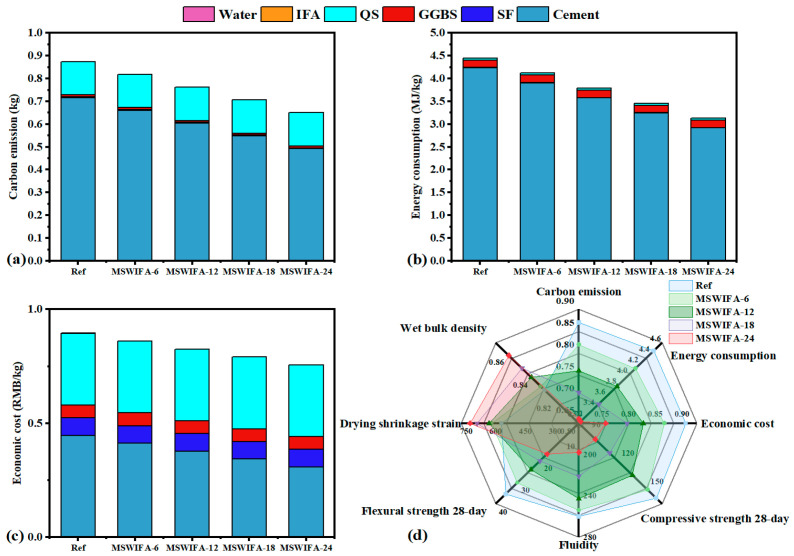
(**a**) Carbon emission, (**b**) energy consumption, (**c**) economic cost, and (**d**) multi-criteria assessment of UHPC with different MSWIFA content.

The comparison of carbon emissions, energy consumption, and economic cost of UHPC with varying MSWIFA content is illustrated in Figure 19a–c. In the reference group, cement accounted for 82.85% of carbon emissions and 95.24% of energy consumption. With increasing MSWIFA content, carbon emissions, energy consumption, and economic costs gradually decreased. Compared to the reference group, MSWIFA-6, MSWIFA-12, MSWIFA-18, and MSWIFA-24 exhibited total carbon emission reductions of 6.72%, 13.03%, 19.55%, and 26.07%, respectively; energy consumption reductions of 7.44%, 14.85%, 22.29%, and 29.69%; and total economic cost reductions of 0.59%, 1.19%, 1.78%, and 2.37%. This reflected the disparity between the carbon neutrality of MSWIFA and the high emission coefficient of cement, indicating a significant synergistic optimization effect on environmental and economic aspects within the material system. It should be noted that the treatment cost of MSWIFA was approximately 5000–6000 RMB per ton [48], thus utilizing MSWIFA as a cement substitute in UHPC offered substantial benefits in energy savings, carbon reduction, and economic efficiency improvement.

In summary, employing MSWIFA to replace cement in UHPC preparation not only facilitated the resource utilization of hazardous waste but also ensured the performance of UHPC, while reducing raw material consumption and significantly lowering carbon emissions. This approach combined favorable economic and environmental advantages, providing robust support for advancing green and low-carbon building development and contributing positive implications for achieving sustainable development goals.

The quantitative analysis utilizing radar charts, as illustrated in Figure 19d, compared the comprehensive performance of Ref, MSWIFA-6, MSWIFA-12, MSWIFA-18, and MSWIFA-24. MSWIFA-12 demonstrated superior comprehensive performance in terms of carbon emissions, energy consumption, economic cost, 28-day compressive strength, 28-day flexural strength, and drying shrinkage rate. As the substitution rate of MSWIFA increased, the mechanical properties of UHPC progressively declined. Conversely, the carbon emissions, energy consumption, and economic cost of UHPC improved with an increasing MSWIFA substitution rate. Consequently, when the substitution rate of MSWIFA was within 24%, UHPC or HPC with satisfactory comprehensive performance could be obtained. The findings of this study not only validated the feasibility of the solid waste substitution strategy in the field of ecological building materials but also provided theoretical support and practical basis for achieving low-carbonization and sustainable development of UHPC.

## 4. Conclusions

This study investigated the feasibility of utilizing MSWIFA as a partial substitute for cement in UHPC, with a focus on its impact on physical properties, mechanical properties, durability, sustainability, and ecological economic benefits. The experimental results lead to the following key conclusions.

(1) Incorporating silica fume alone at ≤6% improves fluidity; at 10% it significantly increases 28-day compressive strength through micro-filler action and C-S-H densification. Ground slag at ≤20% used singly prolongs setting, enhances pumpability and offsets flow loss; at 30% it slightly reduces early strength but surpasses the reference at 56 days. MSWIFA particles exhibit a coarse texture and a large specific surface area. The partial replacement of cement with MSWIFA in UHPC leads to a decrease in fluidity, a reduction in setting time, and an increase in wet packing density.
(2) The substitution of cement with MSWIFA adversely affects the mechanical properties of UHPC. Specimens from the MSWIFA-6 and MSWIFA-12 groups attain 28-day compressive strengths of 150.1 MPa and 134.6 MPa, respectively, alongside flexural strengths of 30.4 MPa and 24.2 MPa at the same curing age. Within the substitution range of 12%, these mechanical properties satisfy the requirements for UHPC. Specimens in the MSWIFA-18 and MSWIFA-24 groups exhibit 28-day compressive strengths of 111.3 MPa and 96.7 MPa, respectively. Within a 24% replacement rate range, their mechanical properties meet the requirements for HPC.
(3) The incorporation of MSWIFA increases the drying shrinkage of UHPC. After immersion in chloride and sulfate solutions, both mass and compressive strength of UHPC exhibit an initial increase followed by a subsequent decrease. The corrosion coefficient demonstrates positive values at 28 days but turns negative at 56 days. The maximum mass loss rate after immersion in both solutions reaches 1.53%, while the maximum corrosion coefficient attains 1.127. At a 24% replacement rate, MSWIFA-UHPC displays excellent resistance to chloride ion penetration and sulfate attack.
(4) This study shows that up to 12 wt% MSWIFA can be incorporated into UHPC. Furthermore, the resulting UHPC maintains a compressive strength above 120 MPa and immobilizes heavy metals to meet both Chinese and EU leaching limits.
(5) The substitution of cement with MSWIFA in UHPC resulted in reductions of 7.42–29.73% in energy consumption, 6.38–25.51% in CO_2_ emissions, and 2.56–15.38% in cost. This MSWIFA replacement strategy demonstrates significant synergistic environmental and economic benefits, facilitating efficient resource utilization and providing a feasible pathway for the green and low-carbon transformation of UHPC.

In summary, the use of MSWIFA as a cementitious material in UHPC offers a viable alternative to conventional cement, meeting concrete performance requirements while delivering environmental benefits and cost savings. This approach enhances resource utilization efficiency, reduces environmental impacts from cement production, and promotes the sustainable development of UHPC. Although the ultra-dense matrix retards ionic transport, the high chloride content in MSWIFA substantially lowers the corrosion threshold of steel reinforcement, potentially triggering rapid pitting corrosion and structural cracking. Consequently, we limit the MSWIFA replacement level to < 12% for reinforced concrete elements and recommend synergistic implementation with corrosion-inhibiting admixtures or epoxy-coated rebars. Furthermore, pre-treatment techniques such as water-washing or high-temperature activation can be employed to remove or immobilize chloride ions in MSWIFA, thereby reducing its adverse impact on concrete durability. In summary, MSWIFA-UHPC provides a viable pathway for high-value utilization of waste incineration fly ash; however, application within steel infrastructure necessitates pre-washing of MSWIFA alongside performance-based design guidelines that integrate chloride binding capacity, carbonation resistance, and heavy metal leaching into a unified durability index.

## Figures and Tables

**Table 1:** Basic properties of cement.

Specific Surface Area (m^2^·kg^−1^)	Soundness	Setting Time (min)		Compressive Strength (MPa)		Flexural Strength (MPa)	
		Initial	Final	7 Days	28 Days	7 Days	28 Days
348	Qualified	195	255	36.6	60.1	5.9	8.4

**Table 2:** Chemical composition of raw material in mass percent (%).

Materials	CaO	Cl	SO_3_	SiO_2_	K_2_O	Na_2_O	MgO	Al_2_O_3_	Fe_2_O_3_	Loss
Cement	56.90	-	2.36	24.30	0.87	0.13	3.01	7.07	2.51	2.85
Silica fume	0.65	-	0.47	95.06	0.27	0.09	0.59	0.25	0.08	2.54
Slag	34.00	-	1.62	34.20	-	-	6.21	17.60	1.01	5.36
MSWIFA	43.06	22.15	7.85	6.08	5.74	5.70	3.50	2.02	1.68	2.22
QS	0.21	0.31	0.35	98.20	0.08	0.05	0.12	0.61	0.14	0.11

**Table 3:** MSWIFA heavy metal leaching concentration and national standard limits.

Heavy Metal Ions	Pb	Zn	Cu	Cr	Cd
Leaching concentration (mg/L)	5.18	13.97	3.42	0.72	1.83
Standard limits for leaching toxicity of hazardous wastes in China (mg/L)	5	100	100	5	1
EU standard limits for leaching toxicity of hazardous wastes (mg/L)	0.25	50	10	0.5 (Cr^6+^)	0.04
MSW landfill control standards (mg/L)	0.25	100	40	0.5	0.15

**Table 4:** Composition of the UHPC samples.

UHPC Sample	Cement (g)	Silica Fume(g)	Slag (g)	MSWIFA (g)	QS (g)	Water (g)	Superplasticizer * (%)
Ref	615.92	84.96	99.12	0	464	144	2
MSWIFA-6	567.92	84.96	99.12	48	464	144	2
MSWIFA-12	519.92	84.96	99.12	96	464	144	2
MSWIFA-18	471.92	84.96	99.12	144	464	144	2
MSWIFA-24	423.92	84.96	99.12	192	464	144	2

* The superplasticizer dosage is expressed as a percentage by mass relative to the total cementitious materials.

**Table 5:** Heavy metal leaching concentrations of UHPC with different MSWIFA content.

Heavy Metal		Pb	Zn	Cu	Cr	Cd
Leaching concentration (mg/L)	MSWIFA-6	0.11	0.12	0.006	0.012	0.002
	MSWIFA-12	0.10	0.98	0.019	0.034	0.007
	MSWIFA-18	0.17	4.34	2.58	0.15	0.70
	MSWIFA-24	0.21	8.55	2.81	0.50	0.80
Standard limits for leaching toxicity of hazardous wastes (mg/L)		5	100	100	5	1
EU standard limits for leaching toxicity of hazardous wastes (mg/L)		0.25	50	10	0.5 (Cr^6+^)	0.04
Control standard for municipal solid waste landfills (mg/L)		0.25	100	40	0.5	0.15

**Table 6:** Energy consumption, carbon emission and economic cost of raw materials.

Materials	Energy Consumption (MJ/kg)	Carbon Emission (kg)	Economic Cost (RMB/kg)
Cement (52.5)	5.5	0.93	0.48
Silica fume	0.06	0.03	0.73
Slag	1.33	0.07	0.18
Quartz sand	0.067	0.233	0.50
MSWIFA	0	0	0
Water	0.01	0.001	0.0028

**Table 7:** Environmental impact and cost comparison of UHPC with different MSWIFA replacement ratios.

UHPC Sample	Energy Consumption (MJ/kg)	Reduction Rates (%)	Carbon Emission (kg)	Reduction Rates (%)	Economic Cost (RMB/kg)	Reduction Rates (%)
Ref	4.45	0.00	0.87	0.00	0.78	0.00
MSWIFA-5	4.12	7.42	0.82	6.38	0.76	2.56
MSWIFA-10	3.79	14.86	0.76	12.76	0.73	6.41
MSWIFA-20	3.46	22.30	0.71	19.14	0.69	11.54
MSWIFA-30	3.13	29.73	0.65	25.51	0.66	15.38

## Data Availability

The original contributions presented in this study are included in the article material. Further inquiries can be directed to the corresponding authors.

## References

[1] Liu F., Liu H., Yang N. (2021). Comparative study of municipal solid waste incinerator fly ash reutilization in China: Environmental and economic performances. Resour. Conserv. Recycl..

[2] Zhang Y., Wang L., Chen L. (2021). Treatment of municipal solid waste incineration fly ash: State-of-the-art technologies and future perspectives. J. Hazard. Mater..

[3] Snellings R., Suraneni P., Skibsted J. (2023). Future and emerging supplementary cementitious materials. Cem. Concr. Res..

[4] Deng X., Xie C., Zhang J. (2024). Techno-economic analysis of municipal solid waste treatment for poly-generation system. Sci. Total Environ..

[5] Wang H., Zhao B., Zhu F. (2023). Study on the reduction of chlorine and heavy metals in municipal solid waste incineration fly ash by organic acid and microwave treatment and the variation of environmental risk of heavy metals. Sci. Total Environ..

[6] Garcia-Lodeiro I., Carcelen-Taboada V., Fernández-Jiménez A. (2016). Manufacture of hybrid cements with fly ash and bottom ash from a municipal solid waste incinerator. Constr. Build. Mater..

[7] Guo X., Shi H., Wu K. (2016). Performance and risk assessment of alinite cement-based materials from municipal solid waste incineration fly ash (MSWIFA). Mater. Struct..

[8] Liu J., Wang Z., Xie G. (2022). Resource utilization of municipal solid waste incineration fly ash-cement and alkali-activated cementitious materials: A review. Sci. Total Environ..

[9] Liu X., Xie X., Liu R. (2023). Manufacture of alkali-activated cementitious materials using municipal solid waste incineration fly ash (MSWIFA): The effect of the Si/Al molar ratio on fresh and hardened properties. Constr. Build. Mater..

[10] Liu J., Hu L., Tang L. (2021). Utilisation of municipal solid waste incinerator (MSWI) fly ash with metakaolin for preparation of alkali-activated cementitious material. J. Hazard. Mater..

[11] Wang B., Ding W., Fan C. (2024). Immobilization properties and reaction mechanism of municipal solid waste incineration fly ash with alkali activation technology. J. Build. Eng..

[12] Xu Q., Shang N., Ko J.H. (2024). Utilization of municipal solid waste incineration (MSWIFA) in geopolymer concrete: A study on compressive strength and leaching characteristics. Materials.

[13] Wong G., Gan M., Fan X. (2021). Co-disposal of municipal solid waste incineration fly ash and bottom slag: A novel method of low temperature melting treatment. J. Hazard. Mater..

[14] Bie R., Chen P., Song X. (2016). Characteristics of municipal solid waste incineration fly ash with cement solidification treatment. J. Energy Inst..

[15] Chen Y., Zheng Y., Zhou Y. (2023). Multi-layered cement-hydrogel composite with high toughness, low thermal conductivity, and self-healing capability. Nat. Commun..

[16] Monteiro P.J.M., Miller S.A., Horvath A. (2017). Towards sustainable concrete. Nat. Mater..

[17] Wang F., Du Y., Jiao D. (2021). Wood-inspired cement with high strength and multifunctionality. Adv. Sci..

[18] Jiang L., Ma Z., Gu Z. (2024). Impregnate carbonation: CO_2_–guided in situ growth of robust superhydrophobic structures on concrete surfaces. Adv. Mater..

[19] Amran M., Makul N., Fediuk R. (2022). Global carbon recoverability experiences from the cement industry. Case Stud. Constr. Mater..

[20] Isteri V., Ohenoja K., Hanein T. (2020). Production and properties of ferrite-rich CSAB cement from metallurgical industry residues. Sci. Total Environ..

[21] Gao P., Zha W., Chu Y. (2025). Calculation model for CO_2_ emissions of blended cement production. J. Clean. Prod..

[22] Fan C., Wang B., Qi Y. (2021). Characteristics and leaching behavior of MSWI fly ash in novel solidification/stabilization binders. Waste Manag..

[23] Tome S., Etoh M.A., Etame J. (2018). Characterization and leachability behaviour of geopolymer cement synthesised from municipal solid waste incinerator fly ash and volcanic ash blends. Recycling.

[24] Lin X., Zhang D., Zhao Z. (2024). High-performance geopolymers with municipal solid waste incineration fly ash: Influence on the mechanical and environmental properties. Buildings.

[25] Tan J., Dan H., Li J. (2022). Use of municipal waste incineration fly ashes (MSWI FA) in metakaolin-based geopolymer. Environ. Sci. Pollut. Res..

[26] Shao Y., Hou H., Wang G. (2016). Characteristics of the stabilized/solidified municipal solid wastes incineration fly ash and the leaching behavior of Cr and Pb. Front. Environ. Sci. Eng..

[27] Holmes R.R., Hart M.L., Kevern J.T. (2017). Heavy metal removal capacity of individual components of permeable reactive concrete. J. Contam. Hydrol..

[28] Amran M., Huang S.S., Onaizi A.M. (2022). Recent trends in ultra-high performance concrete (UHPC): Current status, challenges, and future prospects. Constr. Build. Mater..

[29] Lande I., Thorstensen R.T. (2023). Comprehensive sustainability strategy for the emerging ultra-high-performance concrete (UHPC) industry. Clean. Mater..

[30] Korpa A., Kowald T., Trettin R. (2009). Phase development in normal and ultra high performance cementitious systems by quantitative X-ray analysis and thermoanalytical methods. Cem. Concr. Res..

[31] Bentz D.P., Conway J.T. (2001). Computer modeling of the replacement of “coarse” cement particles by inert fillers in low w/c ratio concretes: Hydration and strength. Cem. Concr. Res..

[32] Abbas S., Nehdi M.L., Saleem M.A. (2016). Ultra-high performance concrete: Mechanical performance, durability, sustainability and implementation challenges. Int. J. Concr. Struct. Mater..

[33] Lv Y., Yang L., Wang J. (2022). Performance of ultra-high-performance concrete incorporating municipal solid waste incineration fly ash. Case Stud. Constr. Mater..

[34] Mao J., Zhou A., Liang Y. (2024). Innovative dual-benefit recycling and sustainable management of municipal solid waste incineration fly ash via ultra-high performance concrete. Sci. Total Environ..

[35] Xing F., Zheng X., Wu D. (2025). Study on synergistic solidification method for municipal solid waste incineration fly ash: Core chemical stabilization and shell physical encapsulation. Case Stud. Constr. Mater..

[36] Partanen J., Backman P., Backman R. (2005). Absorption of HCl by limestone in hot flue gases. Part II: Importance of calcium hydroxychloride. Fuel.

[37] (2021). Test Method for Fluidity of Cement Mortar.

[38] (2009). Standard for Test method of Performance on Building Mortar.

[39] Wong H.H.C., Kwan A.K.H. (2008). Packing density of cementitious materials: Part 1—Measurement using a wet packing method. Mater. Struct..

[40] (2021). Test Method of Cement Mortar Strength (ISO Method).

[41] (2024). Standard for test methods of long-term Performance and Durability of Ordinary Concrete.

[42] (2007). Solid Waste-Extraction Procedure for Leaching Toxicity-Acetic Acid Buffer Solution Method.

[43] (2010). Solid Waste-Extraction Procedure for Leaching Toxicity-Horizontal Vibration Method.

[44] Li Z., Zhang W., Jin H. (2023). Research on the durability and sustainability of an artificial lightweight aggregate concrete made from municipal solid waste incinerator bottom ash (MSWIBA). Constr. Build. Mater..

[45] Fan X., Li Z., Zhang W. (2022). Effects of different supplementary cementitious materials on the performance and environment of eco-friendly mortar prepared from waste incineration bottom ash. Constr. Build. Mater..

[46] Guo P., Meng W., Du J. (2023). Lightweight ultra-high-performance concrete (UHPC) with expanded glass aggregate: Development, characterization, and life-cycle assessment. Constr. Build. Mater..

[47] Wang Q., Chu H., Shi W. (2023). Feasibility of preparing self-compacting mortar via municipal solid waste incineration bottom ash: An experimental study. Arch. Civ. Mech. Eng..

[48] He W., Yang Y., Zhu X. (2025). Experimental research on mechanical and impact properties of ceramsite prepared from secondary aluminum dross and municipal solid waste incineration ash. Sustain. Environ. Res..

